# Defining the Minimal and Optimal Thresholds for Lymph Node Resection and Examination for Intraductal Papillary Mucinous Neoplasm Derived Pancreatic Cancer: A Multicenter Retrospective Analysis

**DOI:** 10.1097/SLA.0000000000006295

**Published:** 2024-04-12

**Authors:** Joseph R. Habib, Ingmar F. Rompen, Sarah R. Kaslow, Mahip Grewal, Paul C. M. Andel, Shuang Zhang, D. Brock Hewitt, Steven M. Cohen, Hjalmar C. van Santvoort, Marc G. Besselink, I. Quintus Molenaar, Jin He, Christopher L. Wolfgang, Ammar A. Javed, Lois A. Daamen

**Affiliations:** 1 New York University Langone Health, Department of Surgery, New York, USA; 2 Regional Academic Cancer Center Utrecht, UMC Utrecht Cancer Center & St. Antonius Hospital Nieuwegein, Department of Surgery, Utrecht, the Netherlands; 3 Amsterdam UMC, location University of Amsterdam, Department of Surgery, Amsterdam, the Netherlands; 4 Cancer Center Amsterdam, the Netherlands; 5 Johns Hopkins Hospital, Department of Surgery, Baltimore, USA; 6 University Medical Center Utrecht, Division of Imaging and Oncology, Utrecht, the Netherlands

**Keywords:** Intraductal Papillary Mucinous Neoplasm, Pancreatic Neoplasms, Pancreatic Cyst, Pancreatic Cancer, Invasive IPMN, Lymph Nodes, N-stage

## Abstract

**Objective:**

To establish minimal and optimal lymphadenectomy thresholds for intraductal papillary mucinous neoplasm (IPMN)-derived pancreatic ductal adenocarcinoma (PDAC) and evaluate their prognostic value.

**Background:**

Current guidelines recommend a minimum of 12–15 lymph nodes (LNs) in PDAC. This is largely based on pancreatic intraepithelial neoplasia (PanIN)-derived PDAC, a biologically distinct entity from IPMN-derived PDAC.

**Methods:**

Multicenter retrospective study including consecutive patients undergoing upfront surgery for IPMN-derived PDAC was conducted. The minimum cut-off for lymphadenectomy was defined as the maximum number of LNs where a significant node positivity difference was observed. Maximally selected log-rank statistic was used to derive the optimal lymphadenectomy cut-off (maximize survival). Kaplan-Meier curves and log-rank tests were used to analyze overall survival (OS) and recurrence-free survival (RFS). Multivariable Cox-regression was used to determine hazard ratios (HR) with 95% confidence intervals (95%CI).

**Results:**

In 341 patients with resected IPMN-derived PDAC, the minimum number of LNs needed to ensure accurate nodal staging was 10 (p=0.040), whereas ≥20 LNs was the optimal number associated with improved OS (80.3 vs. 37.2 months, p<0.001). Optimal lymphadenectomy was associated with improved OS [HR:0.57 (95%CI 0.39–0.83)] and RFS [HR:0.70 (95%CI 0.51–0.97)] on multivariable Cox-regression. On sub-analysis the optimal lymphadenectomy cut-offs for pancreatoduodenectomy, distal pancreatectomy, and total pancreatectomy were 20 (p<0.001), 23 (p=0.160), and 25 (p=0.008).

**Conclusion:**

In IPMN-derived PDAC, lymphadenectomy with at least 10 lymph nodes mitigates under-staging, and at least 20 lymph nodes is associated with the improved survival. Specifically, for pancreatoduodenectomy and total pancreatectomy, 20 and 25 lymph nodes were the optimal cut-offs.

## INTRODUCTION

The poor survival associated with pancreatic ductal adenocarcinoma (PDAC) is largely driven by the high prevalence of advanced stage at diagnosis as well as a predisposition for local and systemic disease progression even after surgical resection.^1, 2^ Several clinicopathologic are associated with recurrence and survival outcomes, improving risk stratification, and guiding personalized therapy decisions.^3, 4^ Of these, disease in the regional lymph nodes consistently demonstrates a strong relationship with survival outcomes, as demonstrated by the American Joint Committee on Cancer (AJCC) staging system.^5^

Surgical oncologic quality metrics emphasize adequate lymphadenectomy to prevent under-staging and ensure clearance of locoregional disease.^6^ Lymph node ratio (LNR), defined as the ratio of positive lymph nodes to total examined lymph nodes, has also been well established as an independent prognostic factor among PDAC patients.^7^ Moreover, an increasing number of harvested lymph nodes is associated with a greater likelihood of node positivity, implying a potential stage migration, and with improved survival.^8, 9^ As such, an adequate lymphadenectomy in patients with PDAC may provide therapeutic benefits in addition to more accurate staging.

Reports vary on the minimum number of lymph nodes for adequate staging. Examination of 12 lymph nodes may be sufficient for accurate AJCC staging for PDAC.^10^ Alternatively, the International Study Group for Pancreatic Surgery (ISGPS) recommends harvesting and examining at least 15 lymph nodes for adequate staging. The ISGPS also highlighted the prognostic importance of lymph node ratio (LNR), which is impacted by both number of positive and harvested lymph nodes.^11^ However, existing studies do not distinguish between the more common pancreatic intraepithelial neoplasia (PanIN)-derived PDACs and intraductal papillary mucinous neoplasm (IPMN)-derived PDAC. Furthermore, considering the growing understanding of differences in biology, disease course, and outcomes between PanIN-derived and IPMN-derived PDAC, a different number of lymph nodes may be appropriate for accurate staging and prognostication in patients with IPMN-derived PDAC.^12, 13^ This multi-institutional study aims to investigate and derive the recommended minimal and optimal number of lymph nodes to be harvested in IPMN-derived PDAC as well as their prognostic value.

## METHODS

### Study Population

Patients with histologically confirmed IPMN-derived PDAC after surgical resection with available data between 2000 and 2021 were identified from four high-volume international centers including the Amsterdam University Medical Center, Johns Hopkins Hospital, New York University Langone Health, and the Regional Academic Cancer Center Utrecht.

Exclusion criteria included concomitant PDAC separate from an adjacent IPMN, gross positive resection margin (R2), missing number of nodes harvested, stage IV disease at diagnosis, and 90-day postoperative mortality. All participating centers obtained local institutional review board approval. The strengthening and reporting of observational studies in epidemiology (STROBE) guidelines were used and the study complied with the 1964 Helsinki Declaration and its later amendments.^14^

### Pathologic Assessment

Pathologists with an expertise in pancreas confirmed histology - invasive carcinoma arising from an IPMN and not a concomitant PDAC lesion. The AJCC TNM system 8^th^ edition was used for staging.^5^ Microscopic evidence of invasive cancer at or within 1 millimeter of the resection margin was defined as R1. LNR was calculated as the number of positive lymph nodes divided by the number of lymph nodes harvested.

### Statistical Analysis

The recommended minimum cut-off for lymph nodes harvested was defined by the maximum number of lymph nodes harvested where a significant difference between node positivity was observed (stage migration) using a χ^2^ or Fisher’s exact test when appropriate. A maximally selected log-rank statistic, analogous to the lowest p-value method, was used to derive the optimal number of lymph nodes examined for overall survival (OS).^15^ Subsequently, patients were stratified according to optimal and suboptimal number of harvested lymph nodes, based on the established cut-off. Categorical variables were displayed as counts and percentages and compared using a χ^2^ or Fisher’s exact test when appropriate. Continuous variables were described as median and interquartile range (IQR) and compared using a Mann-Whitney U test. OS was defined as time between surgical resection and date of death. Recurrence-free survival (RFS) was defined as time between surgical resection and death or recurrence, whichever came first. Patients were censored at the time of last known follow-up visit documented if no event occurred. Kaplan-Meier analysis was performed for all time-to-event analyses, and these were used to derive median OS and RFS with 95% confidence intervals (CI). Log-rank tests were used to compare Kaplan-Meier curves of the overall and stratified cohorts. A backwards selection multivariable Cox-regression analysis, initially including all clinicopathologic factors (age, sex, CA19–9, type of surgery, year of surgery, margin, T-stage, N-stage, optimal lymphadenectomy, histologic subtype, grade, perineural and lymphovascular invasion, and adjuvant chemotherapy), was utilized to select the combination of variables that were most predictive of OS. Additional analysis was performed to investigate the role of LNR (a variable that is representative of both nodal disease and extent of lymphadenectomy) and its impact on OS and RFS. Results were presented as hazard ratios (HR) with corresponding 95% CI. A sub-analysis was then performed by separating patients based on operation: pancreatoduodenectomy (PD), distal pancreatectomy (DP), and total pancreatectomy (TP). Optimal and suboptimal lymphadenectomy cohorts were redefined and analyzed using PD and TP patients based on the newly derived significant cut-offs. A p-value <0.05 was used to determine statistical significance. Statistical analysis was performed using the “R” statistical software (version 4.2.3) using the “MaxStat”, “survminer”, “survival”, and “ggplot2” packages.

## RESULTS

### Study Population

Overall, 341 patients with resected IPMN-derived PDAC were included (Table 1, supplement Figure 1, Supplemental Digital Content 1, http://links.lww.com/SLA/F75). Of these, 189 (55%) were male and 257 (75%) were 65 years or older. Pancreatoduodenectomy (PD) was performed in 209 (61%) patients, while distal pancreatectomy (DP) and total pancreatectomy (TP) were performed in 74 (22%) and 58 (17%) patients, respectively. Furthermore, 192 (56%), 80 (23%), and 69 (20%) patients had N0, N1, and N2 disease, respectively. The median number of positive and harvested lymph nodes was 0 (IQR: 0–3) and 19 (IQR: 14–27), respectively. Adjuvant chemotherapy was administered in 49% of patients, of which 81% were treated with Gemcitabine based regimens. The median number of adjuvant chemotherapy cycles was 6 (IQR: 4–6). Median follow-up for all surviving patients was 40.3 months (IQR: 17.4–72.3).

### Derivation of a Minimal Cut-off to Forego Inadequate Staging

A cut-off of 10 nodes examined was determined to be the minimum number of lymph nodes (p=0.040) that should be harvested for accurate pathological staging. Patients with <10 lymph nodes examined had significantly less nodal disease [node negative: n=17 (77%) and node positive: n=5 (23%)] compared to patients with ≥10 lymph nodes examined [node negative: n=175, (55%) and node positive: n=144 (45%)]. Beyond 10 lymph nodes harvested, no significant stage migration was observed.

Based on tumor size, a significant increase in nodal positivity was observed with increasing tumor size [(<1cm: 91% N0 vs 1–2cm: 66% N0, P=0.009), and (1–2cm: 66% N0 vs 2–3cm: 31% N0, p=0.003)]. Beyond 2cm, no difference in the rate of nodal positivity was observed.

### Derivation of an Optimal Cut-off

**Figure 1.**
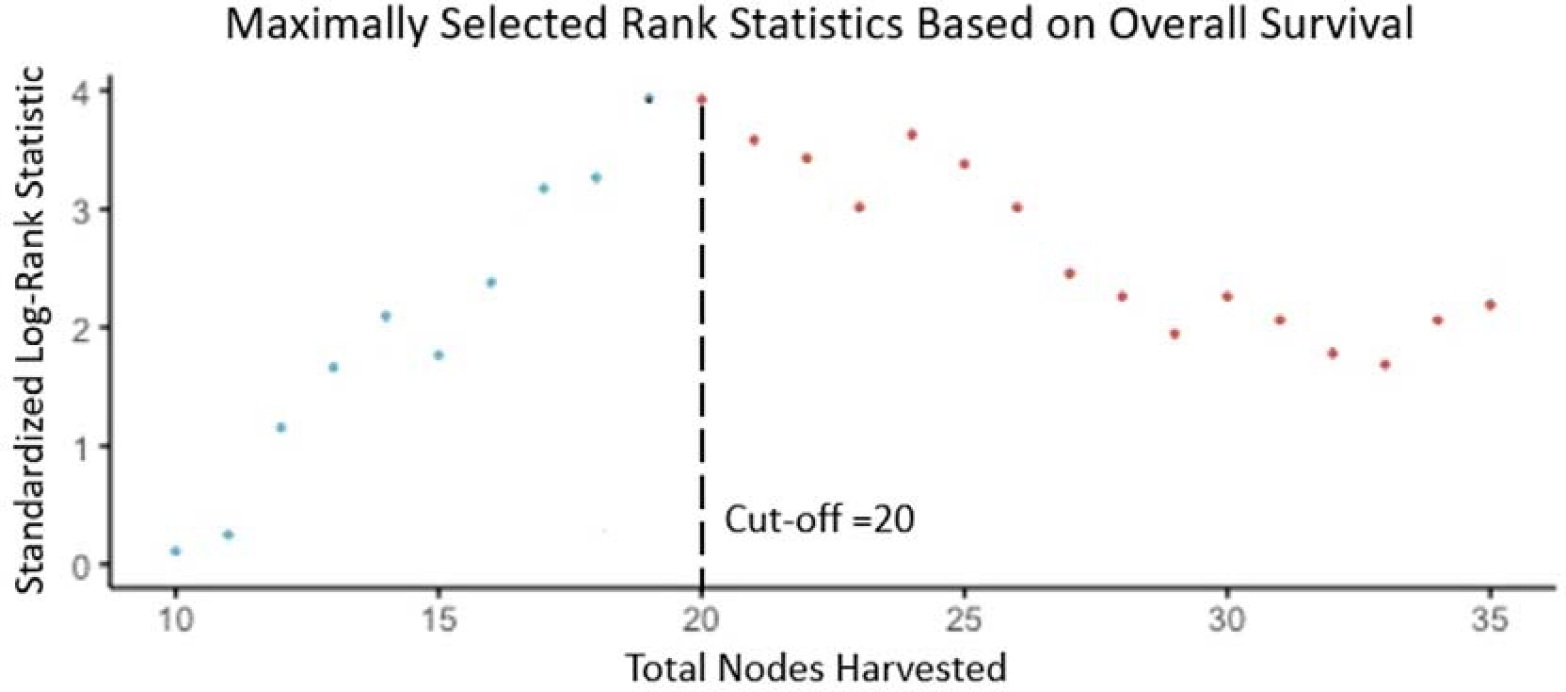
Standardized log-rank statistic for overall survival based to determine optimal cut-off for total lymph nodes harvested

A cut-off of 20 harvested lymph nodes was the optimal cut-off based on survival benefit (Figure 1, p<0.001), which was achieved in 160 out of 341 (47%) patients. Comparisons of baseline characteristics for optimal lymphadenectomy (≥20 harvested lymph nodes) versus suboptimal lymphadenectomy (<20 harvested lymph nodes) are presented in Table 1. Briefly, total pancreatectomy was more common in the optimal lymphadenectomy group (24% vs. 10%, p<0.001). Furthermore, tubular subtype was more common in the suboptimal lymphadenectomy group (84% vs. 71%, p=0.014). There was no difference in American Society of Anesthesiologists (ASA) score (p=0.268), usage of the minimally invasive approach (9% vs. 11%, p=0.576), rate of vascular resections (6% vs. 13%, p=0.100), postoperative complications (40% vs. 49%, p=0.110), receipt of adjuvant chemotherapy (54% vs. 45%, p=0.089), or number of cycles of adjuvant chemotherapy (6 vs 6, p=0.694), while operation duration (mean: 373 vs. 332 minutes, p=0.019) was longer in patients receiving an optimal lymphadenectomy compared to those with a suboptimal lymphadenectomy.

### Survival and Recurrence Outcomes by Optimal Lymphadenectomy

**Figure 2.**
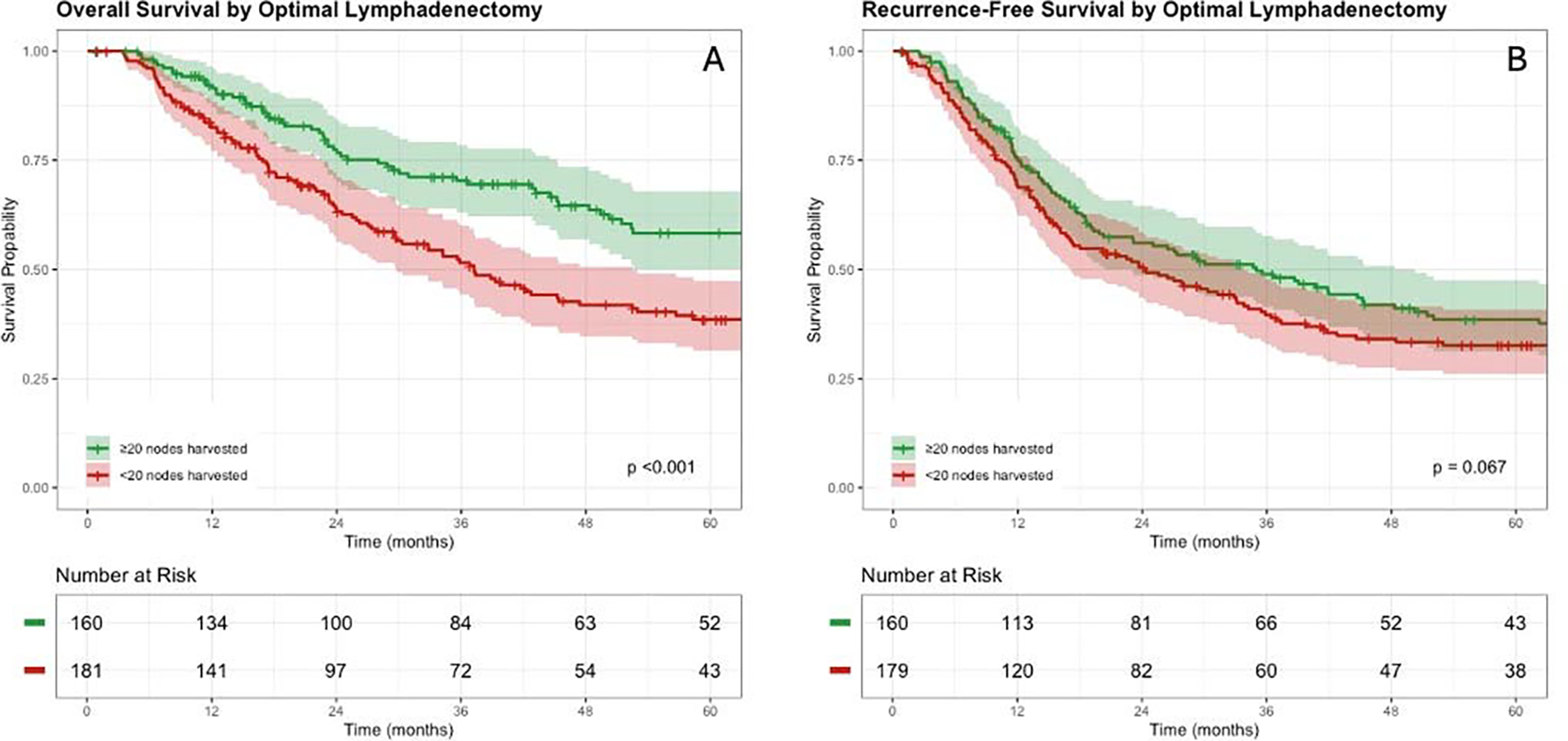
Overall (A) and Recurrence-free (B) survival stratified based on optimal cut-off.

The median OS was 52.5 months (95%CI: 42.8–77.4), while the median RFS was 27.8 months (95%CI: 21.6–35.9). Median OS in the optimal lymphadenectomy group was 80.3 months (95%CI: 66.3–116.0) compared to 37.2 months (95%CI: 29.8–52.5) in the suboptimal lymphadenectomy group (p<0.001) (Figure 2A). Median RFS in the optimal lymphadenectomy group was 35.0 months (95%CI: 23.1–50.2) compared to 24.1 months (95%CI: 17.0–33.8 months) in the suboptimal lymphadenectomy group (p=0.067) (Figure 2B). The distribution of first site of recurrence was locoregional in 29% and 34%, peritoneal in 17% and 11%, systemic in 40% and 36%, while 14% and 19% had multiple sites involved after optimal and suboptimal lymphadenectomy, respectively (p=0.615).

Amongst patients with node negative disease, median OS in the optimal lymphadenectomy group was 113.9 months (95%CI: 93.9–159.0) compared to 85.5 months (95%CI: 67.1–122.0) in the suboptimal group (p=0.140) with a median RFS of 79.9 months (95%CI: 45.2–107.7) and 67.0 months (95%CI: 35.9–97.2 months, p=0.250). Amongst patients with node negative disease, locoregional recurrence was observed in 9% of the optimal lymphadenectomy group compared to 18% in the suboptimal group (p=0.053). No difference was observed in systemic (13% vs. 12%, p=0.870), or peritoneal recurrence (8% vs. 8%, p=0.886).

Amongst patients with node positive disease, median OS in the optimal lymphadenectomy group was 44.5 months (95%CI: 25–77.2) compared to 20.1 months (95%CI: 16.3–27.0) in the suboptimal group (p<0.001) while the difference in RFS was not significant (median RFS 16.1 months (95%CI: 12.0–19.8) vs. 14.0 months (95%CI: 11.6–16.9, p=0.156). Also, there were no significant differences observed for site-specific recurrence rates: locoregional (22% vs. 21%, p=0.807), systemic (30% vs. 37%, p=0.386), and peritoneal (9% vs. 9%, p=0.928).

On pairwise comparisons, patients with N0 disease and a suboptimal lymphadenectomy had similar survival compared to those with an optimal lymphadenectomy and N1 disease (p=0.223). Additionally, patients with N1 disease and a suboptimal lymphadenectomy had similar survival compared to those with N2 disease and an optimal lymphadenectomy (p=0.181). Kaplan-Meier curves and corresponding p-values for pairwise log-rank comparisons are summarized in supplement Figure 2, Supplemental Digital Content 1, http://links.lww.com/SLA/F75 and supplement Table 1, Supplemental Digital Content 1, http://links.lww.com/SLA/F75, respectively.

### Multivariable Regression for Overall and Recurrence-free Survival

**Figure 3.**
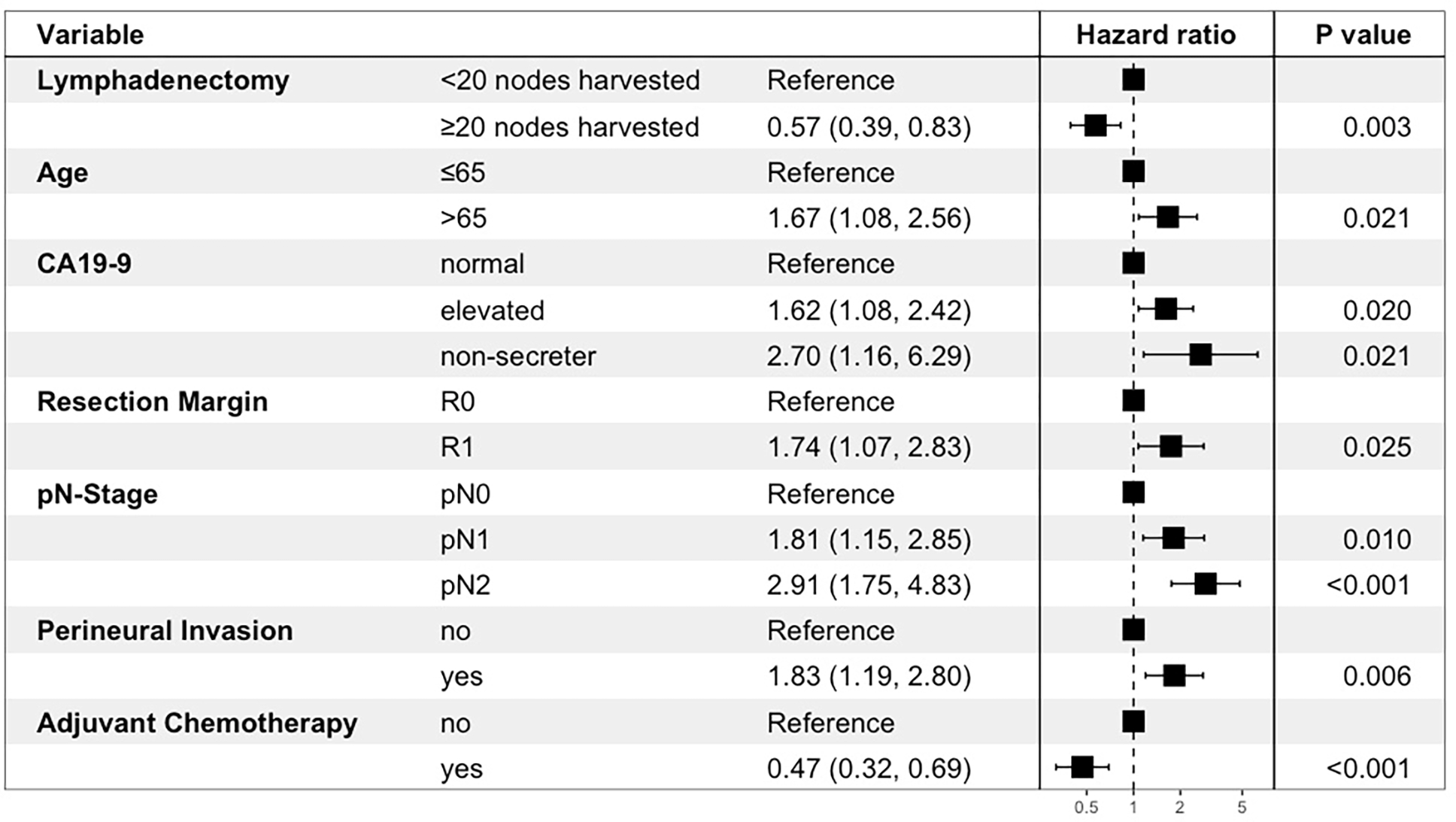
Forrest plot illustrating Cox-regression hazard ratios and 95% confidence intervals for factors associated with overall survival.

**Figure 4.**
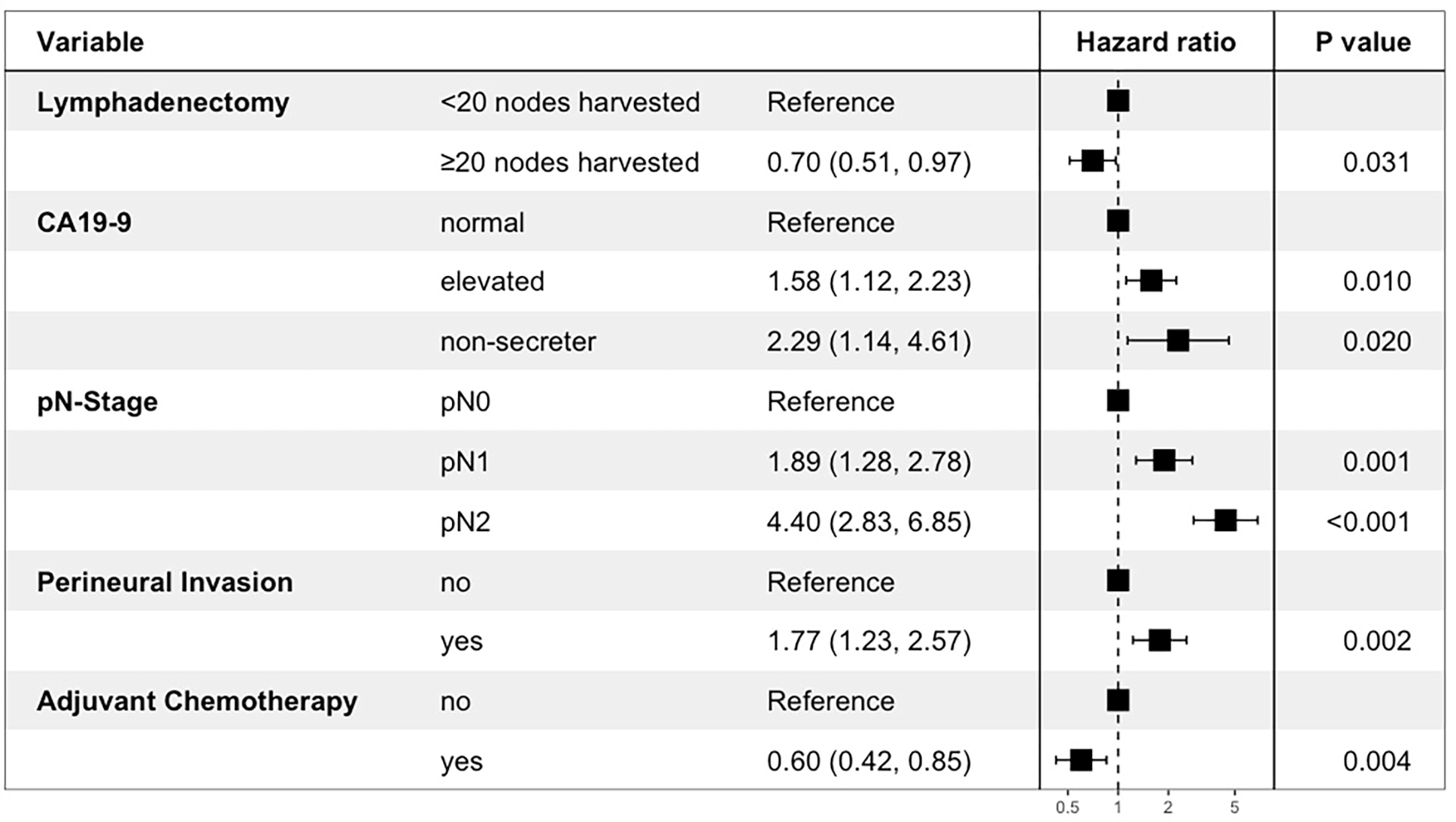
Forrest plot illustrating Cox-regression hazard ratios and 95% confidence intervals for factors associated with recurrence-free survival.

Multivariable Cox-regression analysis showed that optimal lymphadenectomy was associated with improved OS [HR: 0.57 (0.39–0.83), p=0.003] and RFS [HR: 0.70 (0.51–0.97), p=0.031]. Other variables that were associated with OS/RFS in the final model included age, CA19–9, N-stage, perineural invasion, and adjuvant chemotherapy (Figures 3 and 4). Margin status was only significantly associated with RFS. The Harrell’s C statistics for the OS and RFS models were 0.76 and 0.73 respectively.

Additional analysis showed that an increase of 0.1 in LNR was independently associated with worse OS [HR: 1.28 (1.15–1.43), p<0.001] and RFS [HR: 1.40 (1.27–1.54), p<0.001]. Other variables that were associated with OS and RFS in the final model included age, CA19–9, margin, N-stage, perineural invasion, and adjuvant chemotherapy (Supplement Figures 3, Supplemental Digital Content 1, http://links.lww.com/SLA/F75 and 4, Supplemental Digital Content 1, http://links.lww.com/SLA/F75). The Harrell’s C statistic for the OS and RFS models including LNR were 0.75 and 0.74, respectively.

### Sub-analysis by Type of Operation

On sub-analysis for surgical approach, the optimal lymphadenectomy cut-off for PD remained 20 (p<0.001), which was achieved in 95 of 209 (45%) patients undergoing PD. For patients undergoing DP, the optimal cut-off was 23, however, the difference in OS did not reach statistical significance (p=0.160). The optimal cut-off for patients undergoing a TP was 25 (p=0.008), with 28 of the 58 (48%) patients receiving an optimal lymphadenectomy.

In PD and TP patients, where a statistically significant cut-off was detected (n=267, excluding DP), baseline characteristics of optimal (n=123) and suboptimal (n=144) lymphadenectomy are presented in supplement Table 2, Supplemental Digital Content 1, http://links.lww.com/SLA/F75. No differences in minimally invasive approach (p=0.410), vascular resections (p=0.357), duration of operation (p=0.092) or postoperative complications (p=0.356) were observed.

Median OS in the optimal lymphadenectomy group was 93.9 months (95%CI: 70.3–134.9) compared to 30.3 months (95%CI: 24.0–42.7) in the suboptimal lymphadenectomy group (p<0.001) (supplement Figure 5A, Supplemental Digital Content 1, http://links.lww.com/SLA/F75). Median RFS in the optimal lymphadenectomy group was 41.9 months (95%CI: 27.3–77.2 months) compared to 20.1 months (95%CI: 15.2–29.5 months) in the suboptimal lymphadenectomy group (p=0.002) (supplement Figure 5B, Supplemental Digital Content 1, http://links.lww.com/SLA/F75).

Amongst node negative patients, median OS in the optimal lymphadenectomy group was 126.0 months (95%CI: 93.9–200.8) compared to 100.4 months (95%CI: 66.5–124.1) in the suboptimal group (p=0.119) with a median RFS of 98.3 months (95%CI: 52.1–134.9) and 67.1 months (95%CI: 35.4–100.4 months, p=0.101). However, locoregional recurrence was observed less often in the optimal lymphadenectomy group compared to the suboptimal group (6% vs. 19%, p=0.015). The rate of R1 margins within these cohorts similar (p=0.356). There was no difference in systemic (11% vs. 9%, p=0.722), or peritoneal recurrence (7% vs. 4%, p=0.488).

Amongst patients with node positive disease, median OS in the optimal lymphadenectomy group was 44.5 months (95%CI:25.0–171.8) compared to 17.4 months (95%CI: 16.1–24.0) in the suboptimal group (p<0.001).While there was an associated benefit in RFS with optimal lymphadenectomy (median RFS 15.7 months (95%CI:11.5–27.1) vs. 11.7 months (95%CI 9.6–16.1, p=0.011), there were no significant differences observed for site-specific recurrence rates: locoregional (24% vs. 25%, p=0.889), systemic (31% vs. 36%, p=0.579), and peritoneal (12% vs. 6%, p=0.321).

Multivariable Cox-regression analysis on the operation stratified cohorts again validated that optimal lymphadenectomy was associated with improved OS [HR: 0.56 (0.37–0.85), p=0.007, supplement Figure 6, Supplemental Digital Content 1, http://links.lww.com/SLA/F75] and RFS [HR: 0.59 (0.41–0.86), p=0.006, supplement Figure 7, Supplemental Digital Content 1, http://links.lww.com/SLA/F75]. The Harrell’s C statistics for the OS and RFS models were 0.77 and 0.75 respectively.

## DISCUSSION

This first international study focusing on lymphadenectomy in IPMN-derived PDAC determined that a minimum number of 10 lymph nodes should be harvested to prevent under-staging of nodal disease. Furthermore, the optimal number of harvested lymph nodes needed to maximize survival after pancreatectomy is 20. Particularly, the optimal cut-offs for patients undergoing pancreatoduodenectomy was 20 and while it was 25 for total pancreatectomy. Accordingly, we demonstrated that an optimal lymphadenectomy is a robust independent predictor of overall and recurrence-free survival in IPMN-derived PDAC.

Adequate lymphadenectomy is essential for both accurate prognostication when guiding post-operative treatment decision-making and patient counseling and to ensure an adequate oncologic surgical resection. This is especially relevant for IPMN-derived PDAC, since detection of nodal disease is an important determinate for the need for adjuvant chemotherapy.^16^ Currently, for PDAC, guidelines recommend harvesting at least 12–15 lymph nodes.^5, 11^ However, the presence of nodal disease in IPMN-derived PDAC is less common than in PanIN-derived PDAC, and these two cancers are in fact molecularly different entities.^12, 17^ Herein, we aimed to determine a minimal and optimal cut-off for lymphadenectomy unique to patients with IPMN-derived PDAC. Numerous studies have investigated and attempted to identify an optimal number of nodes that should be resected in PanIN-derived PDAC.^18–22^ Javed et al. recommended a higher cut-off of 22 lymph nodes in patients who were treated with neoadjuvant chemotherapy.^19^ The proportion of patients with node positive disease in IPMN-derived PDAC is more similar to those treated with neoadjuvant chemotherapy (40%).^23^ Accordingly, our study recommends a cut-off of 20 lymph nodes for pancreatoduodenectomy and 25 lymph nodes for total pancreatectomy, which is higher than the cut-offs recommended by ISGPS and AJCC.^5, 11^ One study employing the Surveillance and Epidemiology, and End Results (SEER) database found a cut-off of 16 nodes being associated with improved survival in IPMN-derived PDAC in both node positive and negative patients.^24^ Although identifying patients with IPMN-derived PDAC using national databases is not very reliable, our study is more similar to their higher cut-off. However, we found that additional nodes are further associated with improved survival and the present study further differentiated recommendations based on the type of operation.

Like the present study, an increasing number of lymph nodes analyzed has been independently prognostic of improved survival in multiple gastrointestinal malignancies including colon, rectal, pancreas, and esophageal.^9, 25–27^ The rationalization for this finding is debatable. Le Voyer et al. hypothesized that the surgeon may be a variable impacting outcomes.^25^ A surgeon who harvests more lymph nodes performs a more complete excision, possibly removing micrometastatic disease, and is expected to perform a better oncologic resection which may impact survival.^25^ This theory is supported by improved pancreatectomy outcomes at high volume centers with more experienced surgeons.^28^ Moreover, among node negative patients in the operation stratified cohorts, those with a suboptimal number of harvested lymph nodes were more likely to suffer from local disease recurrence compared to those with an optimal lymphadenectomy. This may be explained by improved clearance of disease in the local surgical bed. Although no significant difference was seen in the location of recurrences for node positive patients, an optimal lymphadenectomy in this cohort did significantly extend recurrence-free survival. However, a balance must be found, since an overly aggressive lymphadenectomy has been shown to increase morbidity in one randomized trial.^29^ So, an optimum curve probably exists. However, in the present study, postoperative morbidity was similar between the two cohorts suggesting that an improved oncologic resection may be achieved without impediment of surgical safety with the suggested cut-offs.

A second explanation for improved survival in node negative patients with more harvested nodes is the likelihood of identifying a positive node increases as more nodes are examined. Our study supports a stage migration at a cut-off of 10 nodes harvested as demonstrated by a higher proportion of nodal disease detected in patients with ≥10 lymph nodes examined compared to those with <10 lymph nodes. Wu et al. also comment on a stage migration noted with increased nodes harvested in the SEER database.^24^ Our study recommends a minimum of 10 nodes to be harvested to prevent under-staging of disease. Furthermore, on pairwise comparisons, we found that node negative patients with suboptimal lymphadenectomy exhibited similar survival compared to those with N1 disease and optimal number of lymph nodes harvested. Moreover, patients with N1 disease suboptimal lymphadenectomy had similar survival compared to those with N2 disease and optimal lymphadenectomy. This may further underscore a degree of under-staging in N0 and N1 patients with suboptimal number of lymph nodes harvested (inclusive of those with less than 10 nodes harvested) since their survival outcomes are not different than N1 and N2 patients with an optimal number of lymph nodes harvested.

This study also found LNR, which is a function of both nodal disease and lymphadenectomy, to be a robust and independent predictor of OS and RFS. A binational study between Massachusetts General Hospital and Verona University on 104 patients with IPMN-derived PDAC also reported similar findings observing 5-year RFS rates in patients with LNR = 0, >0 - ≤0.2, and >0.2 of 86.5%, 34.4%, and 11.1%, respectively.^30^ The robustness of LNR as a factor associated with outcomes is also well documented in PanIN-derived PDAC.^6, 22^ Collectively, in both IPMN and PanIN-derived PDAC, lymphadenectomy up to a certain threshold, which directly impacts LNR, appears to have diagnostic and prognostic significance.

Several limitations in the present study should be acknowledged. First, the study period was relatively long and changes in treatment strategies and patient management may have existed. However, this was done to ensure a large study population since a major challenge in investigating IPMNs is their infrequency. Additionally, the years included were not different between the optimal and suboptimal cut-off cohorts. Second, this study involved four international centers where different surgical and pathologic examination practices such as number of examined lymph nodes may be present. Although this may support the generalizability of our findings, future studies should validate these results in a different cohort of patients and further explore the role of resection of specific lymph node stations. Currently, a large proportion of patients undergo resection for an IPMN and are not found to have an invasive component, and hence may not need a minimal or optimal lymphadenectomy. Future investigations on more accurately predicting or assessing for an invasive component (such as intraoperative frozen sections) can help inform the need for a more radical lymphadenectomy. Our study did note an increasing likelihood of nodal positivity in tumors approaching 2cm which may help inform extent of lymphadenectomy. Finally, a strength of the current study is the breakdown of optimal lymphadenectomy based on operation where lymph node yields are different as expected. However, an optimal cut-off was not observed in patients undergoing distal pancreatectomy and future studies should investigate this cohort of patients.

## CONCLUSION

This international study determined that a minimum of 10 lymph nodes should be harvested to prevent under-staging of nodal disease in IPMN-derived PDAC. Furthermore, we demonstrated that the number of harvested nodes is a robust independent predictor of overall and recurrence free survival. Accordingly, the optimal number of harvested lymph nodes needed to maximize survival is 20. In patients undergoing pancreatoduodenectomy, this cut-off remained 20, however 25 lymph nodes were associated with the greatest improvement in survival for patients undergoing total pancreatectomy, which is more than what is currently recommended for PanIN-derived PDAC. The findings of this study could help establish guidelines specific to the management of IPMN-derived PDAC.

## Supplementary Material

Supplement

## Figures and Tables

**Table 1.** Demographics and clinicopathologic data for optimal and suboptimal lymphadenectomy groups using the overall cut-off of 20

Variable	All Patients (N = 341)	Optimal Lymphadenectomy (N = 160)	Suboptimal Lymphadenectomy (N = 181)	P-value

**Male**	189 (55%)	96 (60%)	93 (51%)	0.110

**Age > 65**	269 (75%)	117 (73%)	140 (77%)	0.366

**CA-199** (U/ml)				
Normal (<37)	89 (39%)	45 (40%)	44 (38%)	
Elevated (≥37)	129 (56%)	59 (52%)	70 (60%)	0.071
Non-secreter (<5)	11 (5%)	9 (8%)	2 (2%)	
Unknown	112	47	65	

**Type of Surgery**				
Pancreaticoduodenectomy	209 (61%)	95 (59%)	114 (63%)	
Distal Pancreatectomy	74 (22%)	26 (16%)	48 (27%)	**<0.001**
Total Pancreatectomy	58 (17%)	39 (24%)	19 (10%)	

**Year of Surgery**				
2000–2006	98 (29%)	42 (26%)	56 (31%)	0.269
2007–2013	82 (24%)	35 (22%)	47 (26%)	
2014–2021	161 (47%)	83 (52%)	78 (43%)	

**Minimally Invasive**	35 (10%)	15 (9%)	20 (11%)	0.576
Unknown	5	1	4	

**R1 Margin**	58 (17%)	26 (16%)	32 (18%)	0.726

**pT-stage**				
pT1	127 (38%)	67 (42%)	60 (34%)	
pT2	130 (39%)	59 (37%)	71 (40%)	0.322
pT3/4	79 (24%)	34 (21%)	45 (26%)	
Unknown	5	0	5	

**pN-stage**				
pN0	192 (56%)	93 (58%)	99 (55%)	
pN1	80 (23%)	36 (23%)	44 (24%)	0.816
pN2	69 (20%)	31 (19%)	38 (21%)	

**Tubular**	207 (78%)	94 (71%)	113 (84%)	**0.014**
Unknown	74	28	46	

**Grade of Differentiation**				
Well-moderate	234 (73%)	115 (75%)	119 (72%)	
Poor	85 (27%)	39 (25%)	46 (28%)	0.606
Unknown	22	6	16	

**Perineural Invasion**	185 (57%)	48 (30%)	99 (59%)	0.449
Unknown	14	2	12	

**Lymphovascular Invasion**	106 (32%)	33 (27%)	58 (34%)	0.516
Unknown	11	2	9	

**Adjuvant Chemotherapy**	161 (49%)	82 (54%)	79 (45%)	0.089
Unknown	14	9	5	

## References

[1] Habib JR, Kinny-Koster B, Amini N, Shoucair S, Cameron JL, Thompson ED, Predictors, Patterns, and Timing of Recurrence Provide Insight into the Disease Biology of Invasive Carcinomas Arising in Association with Intraductal Papillary Mucinous Neoplasms. J Gastrointest Surg. 2022;26:2311–20. 35915375 10.1007/s11605-022-05428-4

[2] Hruban RH, Gaida MM, Thompson E, Hong SM, Noe M, Brosens LA, Why is pancreatic cancer so deadly? The pathologist’s view. J Pathol. 2019;248:131–41. 30838636 10.1002/path.5260

[3] Allen PJ, Kuk D, Castillo CF, Basturk O, Wolfgang CL, Cameron JL, Multi-institutional Validation Study of the American Joint Commission on Cancer (8th Edition) Changes for T and N Staging in Patients With Pancreatic Adenocarcinoma. Ann Surg. 2017;265:185–91. 27163957 10.1097/SLA.0000000000001763 PMC5611666

[4] Rompen IF, Levine J, Habib JR, Sereni E, Mughal N, Hewitt DB, Progression of Site-Specific Recurrence of Pancreatic Cancer and Implications for Treatment. Ann Surg. 2023. 10.1097/SLA.0000000000006142 PMC11259998 37870253

[5] Amin MB, Greene FL, Edge SB, Compton CC, Gershenwald JE, Brookland RK, The Eighth Edition AJCC Cancer Staging Manual: Continuing to build a bridge from a population-based to a more “personalized” approach to cancer staging. CA Cancer J Clin. 2017;67:93–9. 28094848 10.3322/caac.21388

[6] Pawlik TM, Gleisner AL, Cameron JL, Winter JM, Assumpcao L, Lillemoe KD, Prognostic relevance of lymph node ratio following pancreaticoduodenectomy for pancreatic cancer. Surgery. 2007;141:610–8. 17462460 10.1016/j.surg.2006.12.013

[7] Karjol U, Chandranath A, Jonnada P, Cherukuru S, Annavarjula V, Morla SA. Lymph Node Ratio as a Prognostic Marker in Pancreatic Cancer Survival: A Systematic Review and Meta-Analysis. Cureus. 2020;12:e9597. 32789099 10.7759/cureus.9597 PMC7417066

[8] Huang L, Jansen L, Balavarca Y, van der Geest L, Lemmens V, Koerkamp BG, Significance of Examined Lymph Node Number in Accurate Staging and Long-term Survival in Resected Stage I-II Pancreatic Cancer-More is Better? A Large International Population-based Cohort Study. Ann Surg. 2021;274:e554–e63. 31425290 10.1097/SLA.0000000000003558

[9] Ghukasyan R, Banerjee S, Childers C, Labora A, McClintick D, Girgis M, Higher Numbers of Examined Lymph Nodes Are Associated with Increased Survival in Resected, Treatment-Naive, Node-Positive Esophageal, Gastric, Pancreatic, and Colon Cancers. J Gastrointest Surg. 2023;27:1197–207. 36854990 10.1007/s11605-023-05617-9

[10] Pu N, Gao S, Beckman R, Ding D, Wright M, Chen Z, Defining a minimum number of examined lymph nodes improves the prognostic value of lymphadenectomy in pancreas ductal adenocarcinoma. HPB (Oxford). 2021;23:575–86. 32900612 10.1016/j.hpb.2020.08.016

[11] Tol JA, Gouma DJ, Bassi C, Dervenis C, Montorsi M, Adham M, Definition of a standard lymphadenectomy in surgery for pancreatic ductal adenocarcinoma: a consensus statement by the International Study Group on Pancreatic Surgery (ISGPS). Surgery. 2014;156:591–600. 25061003 10.1016/j.surg.2014.06.016

[12] Aronsson L, Bengtsson A, Toren W, Andersson R, Ansari D. Intraductal papillary mucinous carcinoma versus pancreatic ductal adenocarcinoma: A systematic review and meta-analysis. Int J Surg. 2019;71:91–9. 31546033 10.1016/j.ijsu.2019.09.014

[13] Ziogas IA, Rodriguez Franco S, Schmoke N, Meguid C, Murphy C, Al-Musawi M, Comparison of Invasive Pancreatic Ductal Adenocarcinoma versus Intraductal Papillary Mucinous Neoplasm: A National Cancer Database Analysis. Cancers (Basel). 2023;15. 36831527 10.3390/cancers15041185 PMC9953895

[14] Vandenbroucke JP, von Elm E, Altman DG, Gotzsche PC, Mulrow CD, Pocock SJ, Strengthening the Reporting of Observational Studies in Epidemiology (STROBE): explanation and elaboration. PLoS Med. 2007;4:e297. 17941715 10.1371/journal.pmed.0040297 PMC2020496

[15] Hothorn TaL Berthold, On the Exact Distribution of Maximally Selected Rank Statistics (February 2002). Science Direct Working Paper No S1574–0358(04)70152–5.

[16] Aronsson L, Marinko S, Ansari D, Andersson R. Adjuvant therapy in invasive intraductal papillary mucinous neoplasm (IPMN) of the pancreas: a systematic review. Ann Transl Med. 2019;7:689. 31930090 10.21037/atm.2019.10.37 PMC6944598

[17] Fritz S, Fernandez-del Castillo C, Mino-Kenudson M, Crippa S, Deshpande V, Lauwers GY, Global genomic analysis of intraductal papillary mucinous neoplasms of the pancreas reveals significant molecular differences compared to ductal adenocarcinoma. Ann Surg. 2009;249:440–7. 19247032 10.1097/SLA.0b013e31819a6e16 PMC3957431

[18] Arrington AK, Price ET, Golisch K, Riall TS. Pancreatic Cancer Lymph Node Resection Revisited: A Novel Calculation of Number of Lymph Nodes Required. J Am Coll Surg. 2019;228:662–9. 30677528 10.1016/j.jamcollsurg.2018.12.031

[19] Javed AA, Ding D, Baig E, Wright MJ, Teinor JA, Mansoor D, Accurate Nodal Staging in Pancreatic Cancer in the Era of Neoadjuvant Therapy. World J Surg. 2022;46:667–77. 34994834 10.1007/s00268-021-06410-y

[20] Malleo G, Maggino L, Ferrone CR, Marchegiani G, Mino-Kenudson M, Capelli P, Number of Examined Lymph Nodes and Nodal Status Assessment in Distal Pancreatectomy for Body/Tail Ductal Adenocarcinoma. Ann Surg. 2019;270:1138–46. 29672406 10.1097/SLA.0000000000002781

[21] Schwarz RE, Smith DD. Extent of lymph node retrieval and pancreatic cancer survival: information from a large US population database. Ann Surg Oncol. 2006;13:1189–200. 16955385 10.1245/s10434-006-9016-x

[22] Slidell MB, Chang DC, Cameron JL, Wolfgang C, Herman JM, Schulick RD, Impact of total lymph node count and lymph node ratio on staging and survival after pancreatectomy for pancreatic adenocarcinoma: a large, population-based analysis. Ann Surg Oncol. 2008;15:165–74. 17896141 10.1245/s10434-007-9587-1

[23] Habib JR, Kinny-Koster B, Bou-Samra P, Alsaad R, Sereni E, Javed AA, Surgical Decision-Making in Pancreatic Ductal Adenocarcinoma: Modeling Prognosis Following Pancreatectomy in the Era of Induction and Neoadjuvant Chemotherapy. Ann Surg. 2023;277:151–8. 33843794 10.1097/SLA.0000000000004915

[24] Wu W, Hong X, Tian R, You L, Dai M, Liao Q, An increased total resected lymph node count benefits survival following pancreas invasive intraductal papillary mucinous neoplasms resection: an analysis using the surveillance, epidemiology, and end result registry database. PLoS One. 2014;9:e107962. 25264746 10.1371/journal.pone.0107962 PMC4179272

[25] Le Voyer TE, Sigurdson ER, Hanlon AL, Mayer RJ, Macdonald JS, Catalano PJ, Colon cancer survival is associated with increasing number of lymph nodes analyzed: a secondary survey of intergroup trial INT-0089. J Clin Oncol. 2003;21:2912–9. 12885809 10.1200/JCO.2003.05.062

[26] Mariette C, Piessen G, Briez N, Triboulet JP. The number of metastatic lymph nodes and the ratio between metastatic and examined lymph nodes are independent prognostic factors in esophageal cancer regardless of neoadjuvant chemoradiation or lymphadenectomy extent. Ann Surg. 2008;247:365–71. 18216546 10.1097/SLA.0b013e31815aaadf

[27] Tepper JE, O’Connell MJ, Niedzwiecki D, Hollis D, Compton C, Benson AB 3rd, Impact of number of nodes retrieved on outcome in patients with rectal cancer. J Clin Oncol. 2001;19:157–63. 11134208 10.1200/JCO.2001.19.1.157

[28] Lieberman MD, Kilburn H, Lindsey M, Brennan MF. Relation of perioperative deaths to hospital volume among patients undergoing pancreatic resection for malignancy. Ann Surg. 1995;222:638–45. 7487211 10.1097/00000658-199511000-00006 PMC1234991

[29] Yeo CJ, Cameron JL, Lillemoe KD, Sohn TA, Campbell KA, Sauter PK, Pancreaticoduodenectomy with or without distal gastrectomy and extended retroperitoneal lymphadenectomy for periampullary adenocarcinoma, part 2: randomized controlled trial evaluating survival, morbidity, and mortality. Ann Surg. 2002;236:355–66; discussion 66–8. 12192322 10.1097/00000658-200209000-00012 PMC1422589

[30] Partelli S, Fernandez-Del Castillo C, Bassi C, Mantovani W, Thayer SP, Crippa S, Invasive intraductal papillary mucinous carcinomas of the pancreas: predictors of survival and the role of lymph node ratio. Ann Surg. 2010;251:477–82. 20142730 10.1097/SLA.0b013e3181cf9155 PMC3135381

