# Supplementary material for: Defining the Minimal and Optimal Thresholds for Lymph Node Resection and Examination for Intraductal Papillary Mucinous Neoplasm–derived Pancreatic Cancer: A Multicenter Retrospective Analysis
Source: Ann Surg. 2024 Apr 12;282(6):1052–9. doi: 10.1097/SLA.0000000000006295 (PMC11470133; doi:10.1097/SLA.0000000000006295)

**SUPPLEMENT**

**SUPPLMENT TABLES**

**Table S1.** Impact of optimal (≥20) or suboptimal (<20) lymphadenectomy stratified by nodal status on survival compared in a pairwise manner.

|  | **Optimal N0** | **Suboptimal N0** | **Optimal N1** | **Suboptimal N1** | **Optimal N2** |
| --- | --- | --- | --- | --- | --- |
| **Suboptimal N0** | 0.175 |  |  |  |  |
| **Optimal N1** | **0.017** | 0.223 |  |  |  |
| **Suboptimal N1** | **<0.001** | **<0.001** | **0.013** |  |  |
| **Optimal N2** | **<0.001** | **0.017** | 0.230 | 0.181 |  |
| **Suboptimal N2** | **<0.001** | **<0.001** | **<0.001** | **0.013** | **0.004** |
| P-values compared using the pairwise log-rank test represent the difference in survival observed between any two select groups | | | | | |

**Table S2.** Demographics and clinicopathologic data for optimal and suboptimal lymphadenectomy groups for pancreatoduodenectomy and total pancreatectomy.

| **Variable** | **Optimal Lymphadenectomy**  **(N = 123)** | **Suboptimal Lymphadenectomy**  **(N = 144)** | **P-value** |
| --- | --- | --- | --- |
| **Male** | 80 (65%) | 74 (51%) | **0.024** |
| **Age > 65** | 87 (71%) | 104 (72%) | 0.788 |
| **ASA**  1  2  3-4  Unknown | 3 (5%)  17 (29%)  38 (66%)  65 | 3 (4%)  37 (49%)  36 (47%)  68 | 0.066 |
| **CA-199** (U/ml) |  |  |  |
| Normal (<37) | 37 (42%) | 34 (37%) |  |
| Elevated (**≥**37) | 45 (51%) | 55 (60%) | 0.144 |
| Non-secreter (<5) | 7 (8%) | 2 (2%) |  |
| Unknown | 34 | 53 |  |
| **Year of Surgery** |  |  | 0.301 |
| 2000-2006f | 36 (29%) | 48 (33%) |  |
| 2007-2013 | 26 (21%) | 38 (26%) |  |
| 2014-2021 | 61 (50%) | 58 (40%) |  |
| **Type of Surgery** |  |  |  |
| Pancreaticoduodenectomy | 95 (77%) | 114 (79%) | 0.766 |
| Total Pancreatectomy      Distal Pancreatectomy | 28 (23%) | 30 (21%) |  |
|  | No optimal cut-off observed | |  |
| **Surgical Approach**  Minimally Invasive  Open  Unknown | 10 (8%)  112 (92%)  1 | 8 (6%)  134 (94%)  2 | 0.410 |
| **R1 Margin** | 21 (17%) | 23 (16%) | 0.809 |
| **pT-stage** |  |  |  |
| pT1 | 51 (41%) | 46 (33%) |  |
| pT2 | 45 (37%) | 59 (42%) | 0.331 |
| pT3/4 | 27 (22%) | 36 (26%) |  |
| Unknown | 0 | 5 |  |
| **pN-stage** |  |  |  |
| pN0 | 72 (59%) | 75 (52%) |  |
| pN1 | 26 (21%) | 35 (24%) | 0.572 |
| pN2 | 25 (20%) | 34 (24%) |  |
| **Tubular**  Unknown | 72 (69%)  18 | 89 (81%)  34 | **0.037** |
| **Grade of Differentiation** |  |  |  |
| Well-moderate | 91 (76%) | 94 (72%) |  |
| Poor | 29 (24%) | 37 (28%) | 0.464 |
| Unknown | 3 | 13 |  |
| **Perineural Invasion** | 62 (50%) | 81 (60%) | 0.112 |
| Unknown | 0 | 9 |  |
| **Lymphovascular Invasion** | 33 (27%) | 47 (34%) | 0.206 |
| Unknown | 9 | 2 |  |
| **Adjuvant Chemotherapy** | 64 (54%) | 62 (45%) | 0.124 |
| Unknown | 5 | 5 |  |

SUPPLEMENT FIGURES

**Figure S1.** Patient contribution by participating institution.


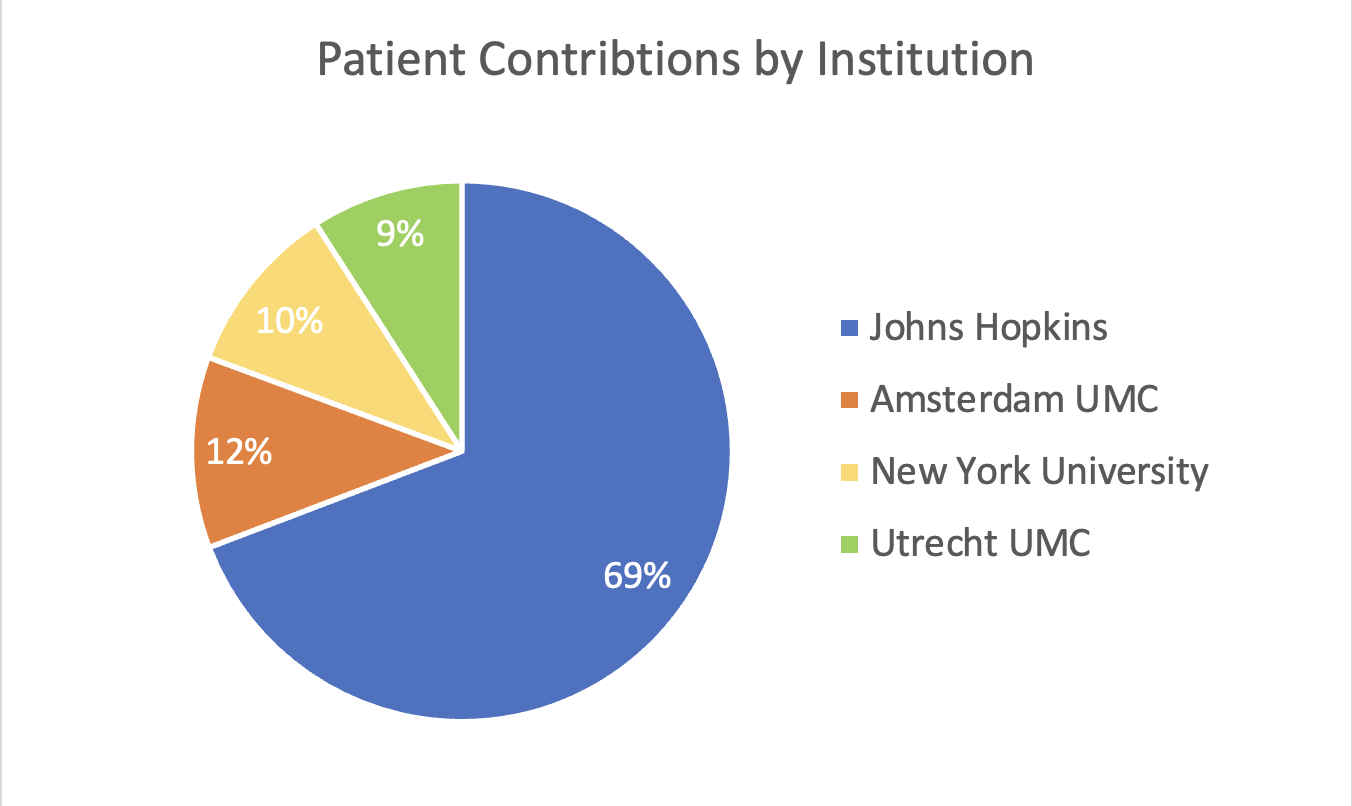


**Figure S2.** Kaplan-Meier survival curves stratified by optimal and suboptimal lymphadenectomy and AJCC N-stage.


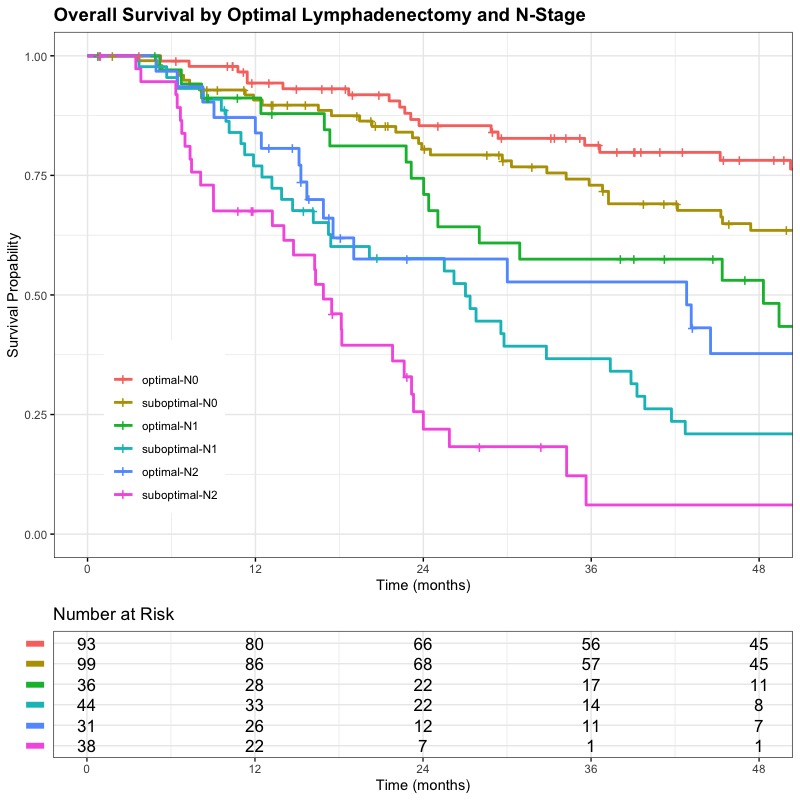


**Figure S3.** Forrest plot illustrating Cox-regression hazard ratios and 95% confidence intervals for factors associated with overall survival using lymph node ratio.


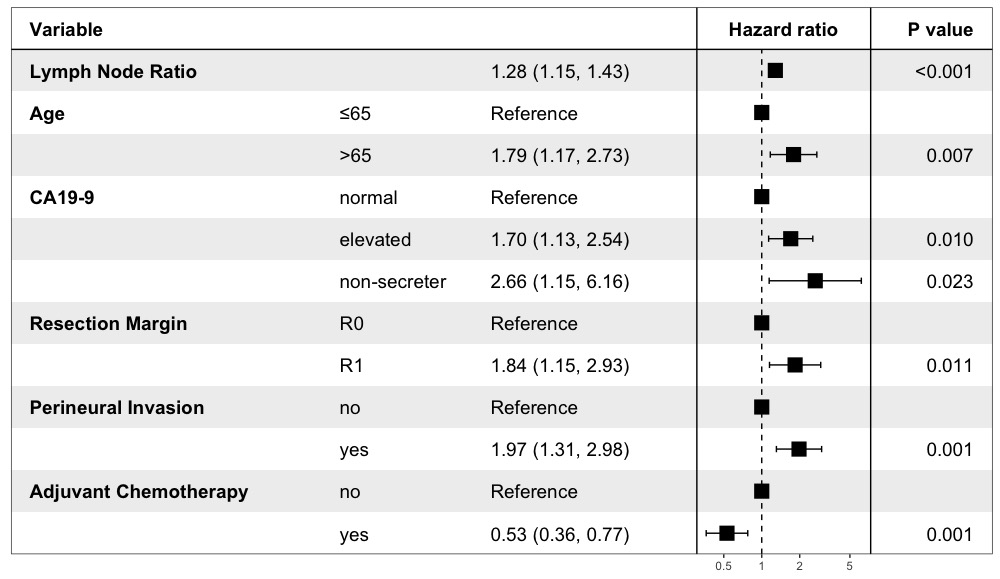


**Figure S4.** Forrest plot illustrating Cox-regression hazard ratios and 95% confidence intervals for factors associated with recurrence-free survival using lymph node ratio.


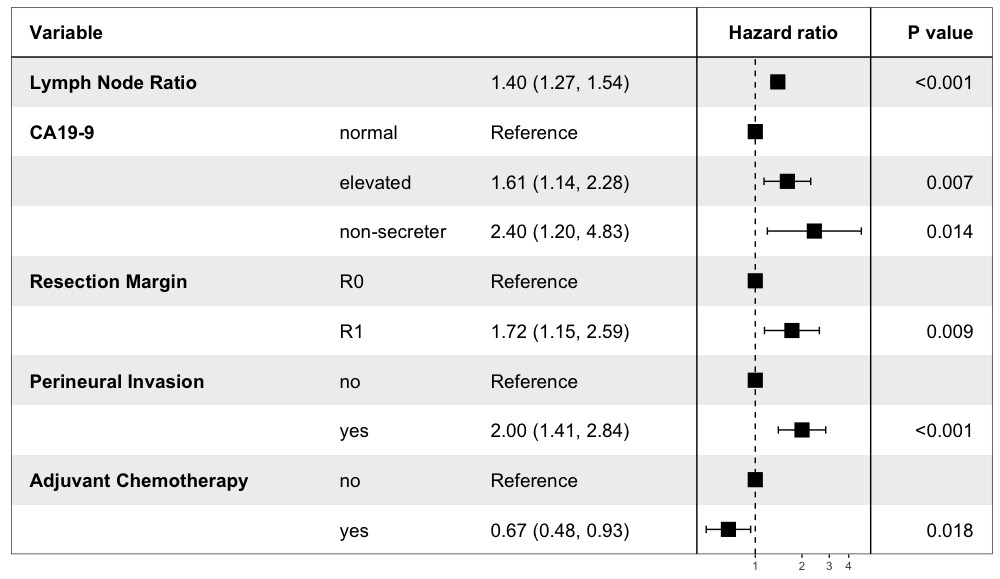


**Figure S5.** Kaplan-Meier survival curves for overall (A) and recurrence-free (B) survival separated by optimal and suboptimal lymphadenectomy using operation stratified cohorts.
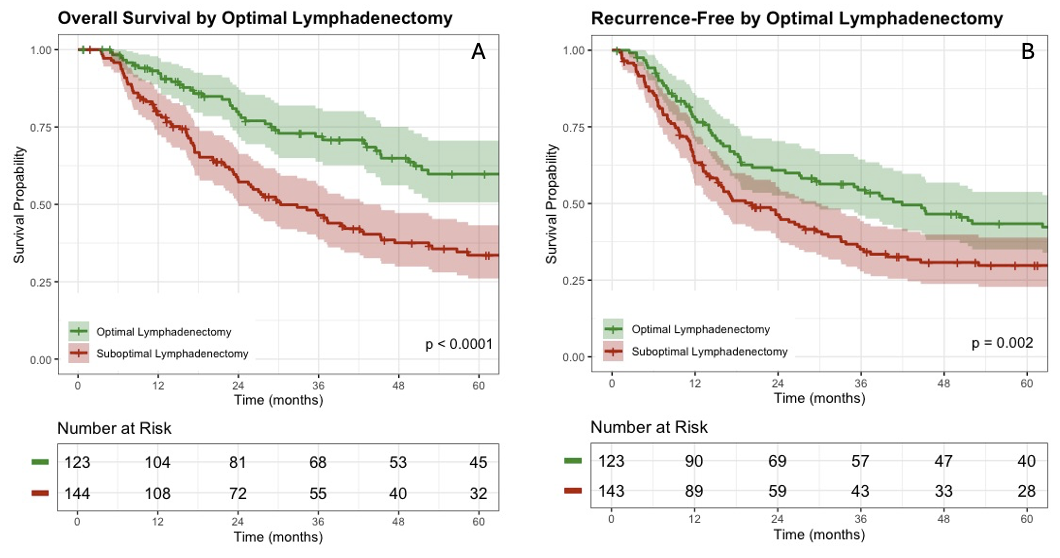


**Figure S6.** Forrest plot illustrating Cox-regression hazard ratios and 95% confidence intervals for factors associated with overall survival using optimal and suboptimal lymphadenectomy in operation stratified cohorts.


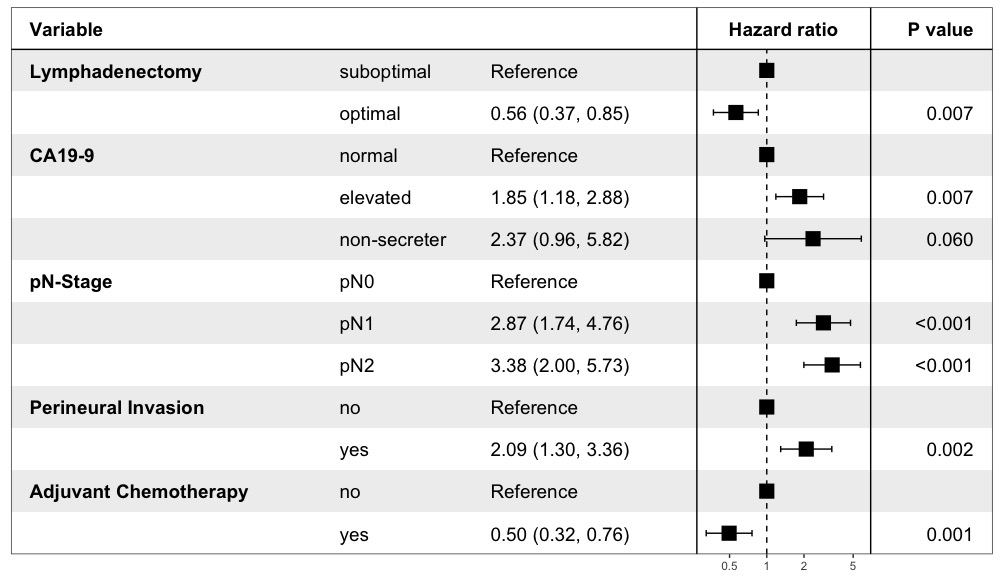


**Figure S7.** Forrest plot illustrating Cox-regression hazard ratios and 95% confidence intervals for factors associated with recurrence-free survival using optimal and suboptimal lymphadenectomy in operation stratified cohorts.


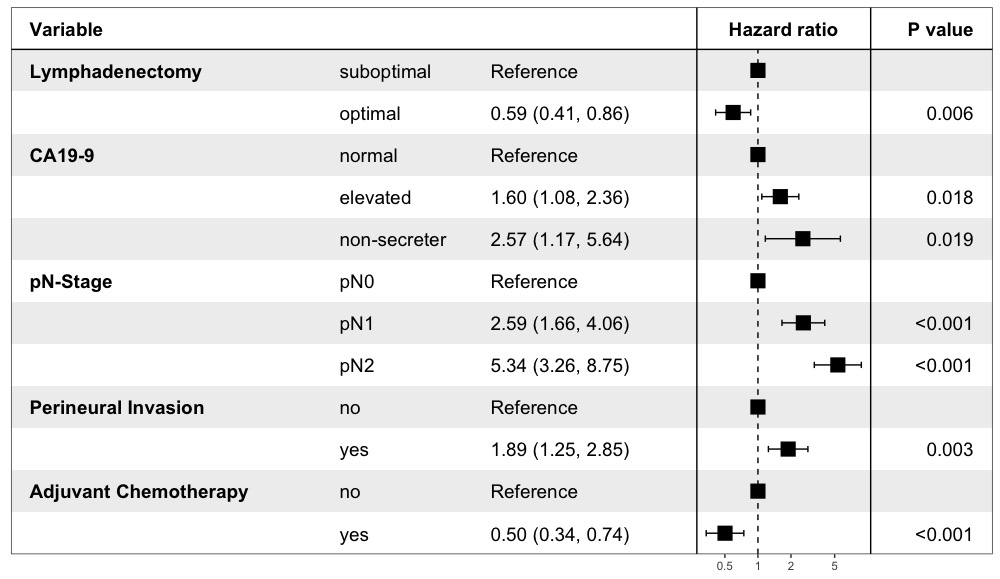

Supplement: Supplementary file 1 [file sla-282-1052-s001.docx]
